# Pharmacokinetic and pharmacodynamic evaluation of rivaroxaban: considerations for the treatment of venous thromboembolism

**DOI:** 10.1186/1477-9560-12-22

**Published:** 2014-10-28

**Authors:** Sebastian Harder

**Affiliations:** Institute of Clinical Pharmacology, Pharmazentrum Frankfurt, University Hospital, Theodor Stern Kai 7, D-60590 Frankfurt am Main, Germany

**Keywords:** Dosing, Pharmacokinetics, Rivaroxaban, Venous thromboembolism treatment

## Abstract

Patients with deep vein thrombosis or pulmonary embolism are recommended to receive anticoagulation for acute treatment and secondary prevention of venous thromboembolism (VTE). Fast-acting direct oral anticoagulants, with or without parenteral heparin, have the potential to replace vitamin K antagonists in this setting. Rivaroxaban, a direct Factor Xa inhibitor, is approved in the European Union and the United States for the single-drug treatment of deep vein thrombosis and pulmonary embolism and the secondary prevention of recurrent VTE in adults. The approved rivaroxaban dose schedule (15 mg twice daily for 3 weeks followed by 20 mg once daily) was derived based on pharmacological data from the clinical development programme to achieve a strong antithrombotic effect in the acute treatment phase and address the need to balance efficacy and bleeding risk for long-term treatment with a once-daily dose in the maintenance phase. Data from dose-ranging studies, pharmacokinetic modelling and randomised phase III trials support the use of this regimen. Other direct oral anticoagulants have also shown favourable efficacy and safety compared with standard treatment, and apixaban (European Union) and dabigatran (European Union and United States) have been approved in this indication. There are practical aspects to rivaroxaban use that must be considered, such as treatment of patients with renal and hepatic impairment, drug–drug interactions, monitoring of effect and management of bleeding. This review discusses the derivation of the VTE treatment regimen for rivaroxaban, summarises the clinical data for rivaroxaban and other direct oral anticoagulants in VTE treatment, and considers the practical aspects of rivaroxaban use in this setting.

## Introduction

Guidelines recommend that patients with deep vein thrombosis (DVT) or pulmonary embolism (PE) should receive anticoagulant treatment, starting with parenteral induction using an agent such as unfractionated heparin (UFH), low molecular weight heparin (LMWH) or fondaparinux [1, 2]. The parenteral anticoagulant is then overlapped with an oral vitamin K antagonist (VKA; e.g. warfarin) until the latter has reached its target anticoagulation level, after which the parenteral agent is discontinued. VKA therapy is continued for as long as the risk of recurrent venous thromboembolism (VTE) outweighs the risk of bleeding [1, 2]. This approach is effective if managed well but does present challenges, both in the acute phase of treatment, when two drugs must be administered together, and over the course of long-term therapy, because of the well-documented limitations of VKAs. The latter include a slow onset and offset of action, multiple drug and food interactions, and variable responses, which necessitate frequent coagulation monitoring and dose adjustments [3].

Fast-acting, single-target, direct oral anticoagulants with more predictable pharmacological properties than VKAs and fewer drug interactions, and that do not require routine coagulation monitoring, have been developed. Four of these anticoagulants (rivaroxaban, apixaban, edoxaban and dabigatran) have been tested against standard therapy in randomised, controlled clinical studies for the treatment of acute VTE and secondary prevention, either alone or after parenteral induction [4–11]. Rivaroxaban, a direct Factor Xa inhibitor, is approved in the European Union and the United States for the single-drug treatment of DVT and PE and the secondary prevention of recurrent VTE in adults. Apixaban, a direct Factor Xa inhibitor, and dabigatran, a direct thrombin inhibitor, are also approved for VTE treatment in the European Union, and dabigatran is also licensed in this indication in the United States.

The approved dose regimen for rivaroxaban in VTE treatment is 15 mg twice daily for the first 3 weeks, and 20 mg once daily thereafter for the specified duration of therapy [12, 13]. This schedule was derived based on the fundamental pharmacological properties of rivaroxaban, data from phase II dose-finding studies [14, 15], pharmacokinetic modelling [16] and randomised phase III clinical trials [4, 5]. This review article summarises the pharmacological and clinical rationale for the single-drug dose regimen of rivaroxaban for VTE treatment, including practical considerations derived from the kinetic and dynamic properties of rivaroxaban.

## Fundamental aspects of rivaroxaban pharmacology

Rivaroxaban is an orally administered anticoagulant that disrupts the action of Factor Xa by selectively but reversibly binding to its active site (dissociation constant of inhibitor [Ki] 0.4 nM) [17, 18]. Inhibition by rivaroxaban occurs whether Factor Xa is freely circulating in plasma, bound in a clot or located in the prothrombinase complex [17, 19]. However, rivaroxaban has no significant effect on the activity of pre-existing thrombin molecules [18].

**Figure 1:**
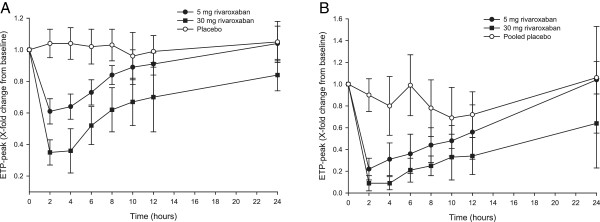
**Effect of rivaroxaban 5 mg and 30 mg, and placebo, on thrombin generation. (A)** ETP peak after activation by collagen (5 μg/ml), **(B)** ETP peak after activation by tissue factor (1.4 nmol). Data are mean relative change from baseline ± standard deviation. The difference between treatments was assessed in this study using the area under the time–effect profile curve; both rivaroxaban doses were significantly different from placebo, but not significantly different from each other. Reproduced with permission from Graff J *et al.*[28]. ETP, endogenous thrombin potential.

A summary of pharmacokinetic information for rivaroxaban and other direct oral anticoagulants is given in Table 1[20–25]. Rivaroxaban has high oral bioavailability (80–100% for a 10 mg dose [12]) and is absorbed rapidly, with maximum plasma concentration (C_max_) reached between 2 and 4 hours and maximum Factor Xa inhibition reached 3 hours after dosing [18, 26]. In a phase I study, suppression of thrombin generation and inhibition of coagulation was evident at 24 hours post-dose (Figure 1) [18, 27, 28].

**Table 1:** **Pharmacokinetic properties of direct oral anticoagulants** [20–25]

	Rivaroxaban	Apixaban	Edoxaban	Dabigatran
Mechanism of action	Direct Factor Xa inhibitor	Direct Factor Xa inhibitor	Direct Factor Xa inhibitor	Direct thrombin inhibitor
Prodrug	No	No	No	Dabigatran etexilate
Oral bioavailability (%)	80–100	~66	~50	6.5
Fraction unbound in plasma (%)	~5–10	13	~41–60	~65–70
t_max_ (h)	2.0–4.0	1.0–3.0	1.0–2.0	1.25–3.0
t_½_ (h)	5–13	8–15	6–11	12–14
Elimination	36% unchanged via active renal secretion; 30% renal excretion of inactive metabolites; 34% hepatobiliary (7% unchanged)	~25% renal; ~75% hepatobiliary	~35–39% renal; ~61–65% hepatobiliary	80% renal; 20% hepatobiliary
Metabolism	CYP3A4, CYP2J2 and CYP-independent mechanisms; P-gp substrate	CYP3A4; P-gp substrate	CYP3A4; P-gp substrate	P-gp substrate

CYP, cytochrome P450; P-gp, P-glycoprotein; t_½_, half-life; t_max_, time to maximum concentration.

Overall, rivaroxaban showed predictable pharmacokinetic and pharmacodynamic properties [18, 26], and a half-life of 5–9 hours in young, healthy subjects and 11–13 hours in elderly subjects [12] (Table 1). No significant accumulation of rivaroxaban occurred with multiple doses in healthy adults [26]. Age, sex and body weight did not affect rivaroxaban clearance to a clinically relevant extent [29, 30], and no significant effect of Caucasian, African-American, Hispanic, Japanese or Chinese ethnicity was noted [12, 31, 32]. Under fasting conditions, exposure was linear and proportional to dose for the 2.5 mg and 10 mg tablets, but at higher doses increases in C_max_ and area under the concentration–time curve (AUC) were dose-proportional but non-linear [33]. Linear dose proportionality was restored by taking the 15 mg and 20 mg tablets with food [12].

Two-thirds of a rivaroxaban dose undergoes metabolic degradation and is excreted in approximately equal proportions via the hepatobiliary route and by the kidneys [12, 20] without evidence of any major or pharmacologically active metabolites circulating in plasma [20]. Metabolic breakdown occurs via cytochrome P450 (CYP) 3A4 and CYP2J2, as well as by CYP-independent mechanisms; rivaroxaban is also a substrate of P-glycoprotein (P-gp) [12, 25]. One-third of the rivaroxaban dose is eliminated in the urine as unchanged drug via active renal secretion [12, 20].

Given the dual renal/metabolic elimination profile of rivaroxaban, renal impairment and the presence of drugs that inhibit metabolic degradation are the main factors that can reduce rivaroxaban clearance and increase exposure. The practical implications of this are further discussed later in the manuscript. Compared with healthy subjects, exposure (AUC) in patients with moderate (creatinine clearance [CrCl] 30–49 ml/min) and severe (CrCl <30 ml/min) renal impairment was increased by 1.5-fold and 1.6-fold, respectively [34], and this led to increased pharmacodynamic activity (Factor Xa inhibition). The overall effect of moderate or severe renal impairment on rivaroxaban pharmacokinetics was considered to be moderate. There are no data for patients with CrCl <15 ml/min [12]. Exposure was increased to a clinically relevant degree in healthy subjects when rivaroxaban was co-administered with strong inhibitors of both CYP3A4 and P-gp, specifically the human immunodeficiency virus (HIV) protease inhibitor ritonavir and the azole-antimycotic ketoconazole, although drugs that were weaker inhibitors of both pathways, or strong inhibitors of only one, had a lesser effect [25]. The anticoagulants enoxaparin and warfarin have been shown to have no relevant effect on the pharmacokinetics of rivaroxaban, but an additive pharmacodynamic effect on Factor Xa inhibition was observed [35, 36].

Rivaroxaban plasma concentrations were similar in healthy subjects and those with mild hepatic impairment (Child–Pugh A), but moderate impairment (Child–Pugh B) led to increases in AUC of 2.27-fold and in C_max_ of 1.27-fold [37]. Enhanced inhibition of Factor Xa activity was evident in these subjects [37], meaning that a clinically relevant increase in bleeding risk would be expected in patients with moderate hepatic impairment [12].

## Derivation of a rivaroxaban dose schedule for venous thromboembolism treatment

### Clinical and pharmacological considerations

**Figure 2:**
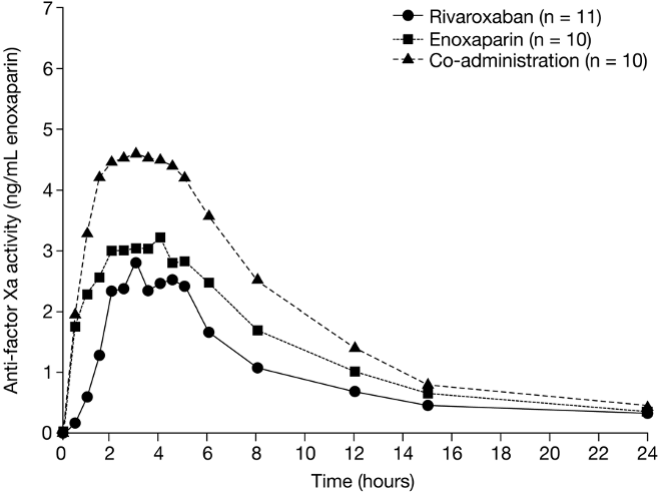
**Anti-Factor Xa activity after administration of oral rivaroxaban and subcutaneous enoxaparin alone and in combination.** Rivaroxaban 10 mg, enoxaparin 40 mg. Median values in healthy subjects. Reproduced with permission from Kubitza D *et al.* [35].

Data from phase III clinical studies of other anticoagulants for VTE treatment suggest that the risk of recurrent VTE is highest during the period following an initial event. In two randomised, phase III studies of acute VTE treatment with the direct thrombin inhibitor ximelagatran and the indirect Factor Xa inhibitor idraparinux, a high risk of recurrent venous thromboembolic events was evident in the first 4 weeks after randomisation [38, 39]. Therefore, if a single oral agent is to replace the standard dual-drug approach, it must exert a rapid effect. In healthy subjects, a 10 mg oral dose of rivaroxaban had a similarly quick onset of action to 40 mg subcutaneous enoxaparin, with peak Factor Xa inhibition occurring for both drugs approximately 3 hours after administration (corresponding to the expected time of rivaroxaban C_max_), indicating that rivaroxaban would be appropriate from the start of therapy without the need for parenteral bridging (Figure 2) [35].

For long-term treatment, the predictable pharmacokinetic and pharmacodynamic profiles of rivaroxaban across patient populations, as well as a lack of food restrictions and relatively few drug interactions, contrast with the variable pharmacology of VKAs and provide the opportunity to employ fixed doses without the need for routine coagulation monitoring [3]. Furthermore, because the pharmacodynamic effects and inhibition of thrombin generation remained evident 24 hours after dosing in phase I studies [18, 28], once-daily schedules for rivaroxaban are feasible. Recent data from an analysis of United States healthcare claims show that once-daily regimens for VTE treatment are associated with better adherence to therapy than twice-daily dosing schedules [40].

### Determination of the rivaroxaban dose schedule in the acute treatment phase

Rivaroxaban was initially evaluated in the phase II, Oral Direct Factor Xa Inhibitor BAY 59–7939 in Patients With Acute Symptomatic Deep-Vein Thrombosis (ODIXa-DVT) dose-finding study [14]. Adult patients with ultrasound-confirmed acute DVT but without PE were randomised to receive rivaroxaban or the LMWH enoxaparin 1 mg/kg twice daily for at least 5 days plus dose-adjusted VKA (either warfarin, phenprocoumon or acenocoumarol) for 12 weeks. Rivaroxaban was dosed at 10, 20 or 30 mg twice daily, or 40 mg once daily. The primary efficacy outcome was the proportion of patients with an improvement in thrombotic burden, which was defined as a reduction in the thrombus score of at least four points evaluated by ultrasound, without confirmed, symptomatic worsening or recurrence of DVT, confirmed symptomatic PE or VTE-related death, at a mean 21 days from the start of treatment (range 18–26 days). The principal safety outcome was the incidence of major bleeding occurring during the 12-week treatment period or up to 2 days after the last anticoagulant dose [14].

For the primary efficacy endpoint, 45.9% of patients who received enoxaparin/VKA had an improvement in thrombotic burden, compared with 53.0%, 59.2%, 56.9% and 43.8% of patients who received rivaroxaban 10, 20 and 30 mg twice daily, and 40 mg once daily, respectively. Major bleeding did not occur in any patients treated with enoxaparin/VKA but was recorded in 1.7%, 1.7%, 3.3% and 1.7%, respectively, of rivaroxaban recipients [14]. Overall, the rivaroxaban twice-daily doses appeared to offer a greater antithrombotic effect than standard therapy in the acute phase, at the cost of a small increase in major bleeding that was lowest with the rivaroxaban 10 mg and 20 mg twice-daily doses.

### Determination of the rivaroxaban dose schedule for long-term treatment

A second, longer-term phase II dose-finding study (EINSTEIN DVT) was also conducted to evaluate the 3-month efficacy and safety of several rivaroxaban doses against enoxaparin/VKA for the prevention of recurrent VTE in patients with confirmed DVT [15]. Patients were randomised to receive rivaroxaban 20, 30 or 40 mg once daily, or 5 days of heparin (UFH or a LMWH [tinzaparin or enoxaparin]) followed by a VKA (warfarin, phenprocoumon, acenocoumarol or fluindione). Treatment was given for 12 weeks. The primary efficacy outcome was the incidence of the composite of symptomatic recurrent DVT, symptomatic fatal or non-fatal PE, and asymptomatic deterioration in thrombotic burden (detected by ultrasound and perfusion lung scanning) at Day 84. The principal safety outcome was the incidence of major plus non-major clinically relevant bleeding occurring up to 2 days after the last anticoagulant dose [15].

The primary efficacy outcome occurred in 6.1%, 5.4% and 6.6% of patients who received rivaroxaban 20, 30 and 40 mg once daily, respectively, compared with 9.9% of patients who received standard therapy. Major or non-major clinically relevant bleeding occurred in 5.9%, 6.0% and 2.2% of patients receiving rivaroxaban, respectively, and in 8.8% of those given standard therapy [15]. There were three cases of major bleeding in the rivaroxaban group and two in the standard therapy group. Based on these outcomes, antithrombotic efficacy with a 20 mg once-daily dose appeared similar to that of the higher doses tested and provided similar efficacy and slightly lower rates of major or non-major clinically relevant bleeding compared with standard therapy [15].

## Pharmacokinetic validation of the rivaroxaban dose regimen

Based on these phase II studies, a rivaroxaban dose regimen of 15 mg twice daily for the first 3 weeks, followed by 20 mg once daily for the remainder of treatment, was proposed for further investigation in phase III studies. This schedule was expected to balance the need for a strong antithrombotic effect in the acute phase, taking into account the risk of bleeding, with the lowest effective once-daily dose for long-term secondary prevention [14, 15].

Data were collected from patients who took part in the two phase II studies in order to create a population-based model of the pharmacokinetics of rivaroxaban in patients with acute DVT [16]. A total of 4634 rivaroxaban plasma samples from 870 patients were collected and input into the model, which was based on previous parameters used for healthy subjects and patients undergoing hip or knee replacement surgery [41–43]. The model showed that the pharmacokinetics of rivaroxaban were predictable across the range of rivaroxaban doses tested (total daily doses of 20–60 mg), and the overall variability between individuals was moderate and consistent over the 12 weeks of treatment [16].

**Figure 3:**
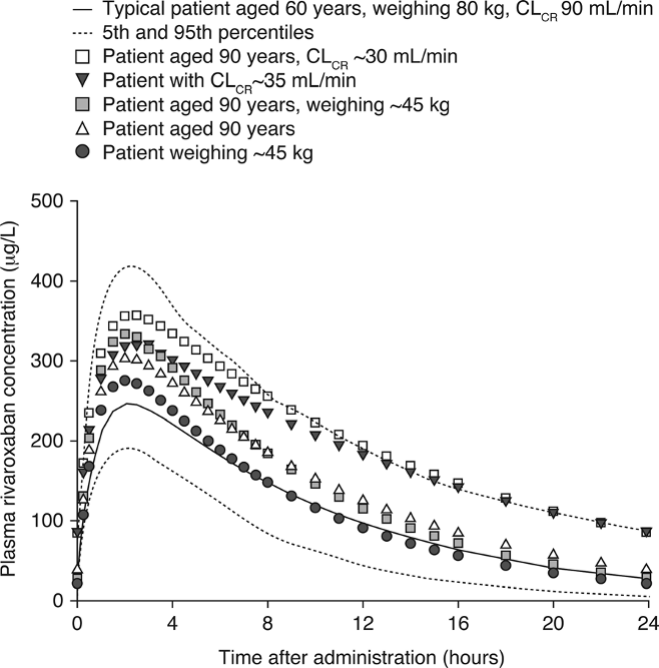
**Predicted plasma rivaroxaban concentration–time profiles.** ‘Typical’ patients and those with extremes of age, renal function and body weight receiving rivaroxaban 20 mg once daily. Reproduced with permission from Mueck W *et al.* [16]. CL_CR_, creatinine clearance.

Variations in age and renal function affected rivaroxaban pharmacokinetics in an anticipated manner, but other demographic factors, such as age and sex, had a minimal effect. When the model was used to simulate plasma rivaroxaban concentration–time profiles after a 20 mg once-daily dose in patients who would be expected to be at high risk of recurrent VTE and/or bleeding, older age and renal impairment had a moderate effect on rivaroxaban exposure, whereas the influence of low body weight was small. Rivaroxaban C_max_ remained within the range of the mean values for the overall population, suggesting that dose reduction would not be necessary in these vulnerable patients (Figure 3) [16].

**Figure 4:**
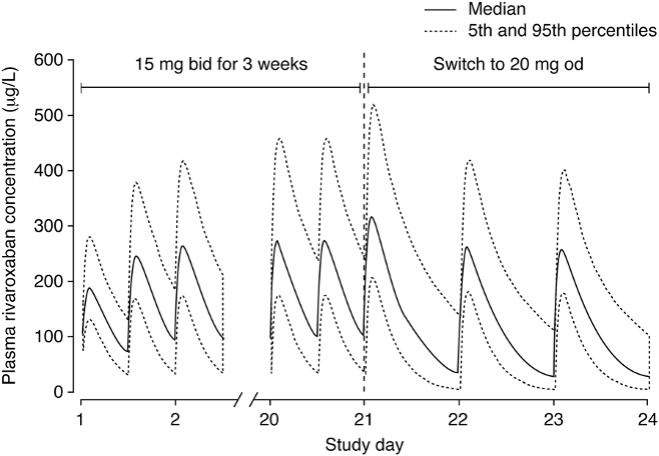
Simulated plasma concentrations of rivaroxaban with the venous thromboembolism treatment regimen*. Reproduced with permission from Mueck W *et al.* [16]. bid, twice daily; od, once daily. *15 mg bid for 3 weeks, followed by 20 mg od.

As in healthy volunteers [41], the C_max_ of rivaroxaban was reached between 2 and 4 hours after dosing, regardless of once- or twice-daily administration [16]. Compared with twice-daily dosing, once-daily rivaroxaban doses led to a higher C_max_ and a lower trough concentration, but the differences were not significant based on the 95% confidence level. Simulation of the proposed rivaroxaban VTE treatment regimen (15 mg twice daily for 3 weeks followed by 20 mg once daily) indicated no substantial fluctuation in C_max_ during the transition from twice-daily to once-daily dosing (Figure 4) [16].

## Randomised phase III studies of rivaroxaban for treatment of venous thromboembolism

The proposed rivaroxaban VTE treatment dose schedule was taken forward to the phase III EINSTEIN programme, which comprised three randomised clinical studies. Two acute treatment trials, EINSTEIN DVT and EINSTEIN PE [4, 5], were complemented by a third study of extended treatment, EINSTEIN EXT [4]. In total, 8282 patients were randomised in EINSTEIN DVT and EINSTEIN PE and a further 1197 were involved in EINSTEIN EXT. Overall, the proportion of patients with unprovoked VTE was reflective of that seen in clinical practice (approximately 63% in EINSTEIN DVT and EINSTEIN PE and 74% in EINSTEIN EXT). Among those with known risk factors for VTE, previous VTE (~19%), recent surgery or trauma (~18%) and immobilisation (~16%) were important in EINSTEIN DVT and EINSTEIN PE; previous VTE (~16%) and immobilisation (~14%) were also prominent characteristics in EINSTEIN EXT. There was a small but relevant proportion of patients with active cancer in all studies (~5%) [4, 5].

EINSTEIN DVT and EINSTEIN PE followed the same study design: patients were randomised to receive either rivaroxaban 15 mg twice daily for 3 weeks, followed by 20 mg once daily, or enoxaparin 1 mg/kg twice daily overlapping with dose-adjusted VKA (warfarin or acenocoumarol) [4, 5]. For the latter, enoxaparin was discontinued after a minimum of 5 days once the international normalised ratio (INR) remained within the target range of 2.0–3.0 for at least 2 days, as per guidelines [1, 2]. EINSTEIN DVT recruited patients with symptomatic, acute DVT confirmed by imaging, but excluded patients who had PE [4]. Conversely, patients included in EINSTEIN PE had confirmed PE with or without DVT [5]; patients with PE who were haemodynamically unstable, who should receive thrombolytic therapy according to guidelines [1, 2], were excluded. Treatment was continued for 3, 6 or 12 months based on clinical evaluation of each patient’s risk of recurrent VTE and bleeding. The primary efficacy outcome in both studies was the incidence of symptomatic, recurrent VTE, and the principal safety outcome was major plus non-major clinically relevant bleeding [4, 5].

In both EINSTEIN DVT and EINSTEIN PE, single-drug rivaroxaban was non-inferior to dual-drug standard therapy for the prevention of recurrent VTE (p < 0.001 and p = 0.003, respectively, for non-inferiority) (Table 2), with a similar incidence of clinically relevant bleeding [4, 5]. Notably, in EINSTEIN PE, there was a 51% relative risk reduction (RRR) in the incidence of major bleeding compared with the enoxaparin/VKA arm [5]. In a pooled analysis of the overall VTE population from the two studies, rivaroxaban remained non-inferior to standard therapy for efficacy and was associated with a significant 46% RRR in the incidence of major bleeding [44]. This led to a 33% relative risk improvement in net clinical benefit (the composite of VTE and major bleeding) with rivaroxaban compared with enoxaparin/VKA [44]. The greater patient numbers afforded by pooling the data also allowed for a more detailed investigation of important patient subgroups that are at increased risk of recurrent VTE and/or bleeding. These included fragile patients (those who were aged >75 years or had CrCl <50 ml/min or body weight ≤50 kg), patients with cancer and those with previous VTE. Rivaroxaban remained non-inferior to standard therapy in these patient groups with a consistent safety profile, and led to a significant 73% RRR in major bleeding in fragile patients [44].

**Table 2:** **Summary of phase III studies of direct oral anticoagulants for the acute treatment of VTE** [4–9]

Drug	Study	Indication	Regimen	Dose schedule	Comparator	Efficacy	Safety
Rivaroxaban	EINSTEIN DVT	Patients with DVT without PE	Single drug	15 mg bid for 3 weeks then 20 mg od for 3, 6 or 12 months	Standard enoxaparin for ≥5 days overlapping with and transitioning to VKA once INR ≥2.0 on 2 consecutive days; thereafter VKA dose adjusted to maintain INR 2.0–3.0	Recurrent VTE: rivaroxaban non-inferior to standard therapy (2.1% vs. 3.0%; HR = 0.68; p < 0.001 for non-inferiority)	Clinically relevant bleeding: similar incidence (8.1% vs. 8.1%; HR = 0.97; p = 0.77)
							Major bleeding: similar incidence (0.8% vs. 1.2%; HR = 0.65; p = 0.21)
Rivaroxaban	EINSTEIN PE	Patients with PE with or without DVT	Single drug	15 mg bid for 3 weeks then 20 mg od for 3, 6 or 12 months	Standard enoxaparin for ≥5 days overlapping with and transitioning to VKA once INR ≥2.0 on 2 consecutive days; thereafter VKA dose adjusted to maintain INR 2.0–3.0	Recurrent VTE: rivaroxaban non-inferior to standard therapy (2.1% vs. 1.8%; HR = 1.12; p = 0.003 for non-inferiority)	Clinically relevant bleeding: similar incidence (10.3% vs. 11.4%; HR = 0.90; p = 0.23)
							Major bleeding: significant reduction with rivaroxaban vs. standard therapy (1.1% vs. 2.2%; HR = 0.49; p = 0.003)
Rivaroxaban	EINSTEIN DVT and PE pooled	Patients with DVT and/or PE	Single drug	15 mg bid for 3 weeks then 20 mg od for 3, 6 or 12 months	Standard enoxaparin for ≥5 days overlapping with and transitioning to VKA once INR ≥2.0 on 2 consecutive days; thereafter VKA dose adjusted to maintain INR 2.0–3.0	Recurrent VTE: rivaroxaban non-inferior to standard therapy (2.1% vs. 2.3%; HR = 0.89; p < 0.001 for non-inferiority)	Clinically relevant bleeding: similar incidence (9.4% vs. 10.0%; HR = 0.93; p = 0.27)
							Major bleeding: significant reduction with rivaroxaban vs. standard therapy (1.0% vs. 1.7%; HR = 0.54; p = 0.002)
Apixaban	AMPLIFY	Patients with DVT and/or PE	Single drug	10 mg bid for 7 days then 5 mg bid for 6 months	Standard enoxaparin for ≥5 days overlapping with and transitioning to warfarin once INR ≥2.0 on 2 consecutive days; thereafter VKA dose adjusted to maintain INR 2.0–3.0	Recurrent VTE: apixaban non-inferior to standard therapy (2.3% vs. 2.7%; RR = 0.84; p < 0.001 for non-inferiority)	Clinically relevant bleeding: significant reduction with apixaban vs. standard therapy (4.3% vs. 9.7%; RR = 0.44; p < 0.001)
							Major bleeding: significant reduction with apixaban vs. standard therapy (0.6% vs. 1.8%; RR = 0.31; p < 0.001)
Edoxaban	Hokusai-VTE	Patients with DVT and/or PE	Dual drug	60 mg^a^ od for 3–12 months after standard heparin induction	Standard heparin for ≥5 days overlapping with and transitioning to warfarin (INR 2.0–3.0)	Recurrent VTE: edoxaban non-inferior to standard therapy (3.2% vs. 3.5%; HR = 0.89; p < 0.001 for non-inferiority)	Clinically relevant bleeding: significant reduction with edoxaban vs. standard therapy (8.5% vs. 10.3%; HR = 0.81; p = 0.004)
							Major bleeding: similar incidence (1.4% vs. 1.6%; HR = 0.84; p = 0.35)
Dabigatran	RE-COVER^b^	Patients with DVT and/or PE	Dual drug	150 mg bid for 6 months after heparin induction	Standard heparin for ≥5 days overlapping with and transitioning to warfarin once INR ≥2.0 on 2 consecutive days; thereafter VKA dose adjusted to maintain INR 2.0–3.0	Recurrent VTE: dabigatran non-inferior to standard therapy (2.4% vs. 2.1%; HR = 1.10; p < 0.001 for non-inferiority)	Clinically relevant bleeding: significant reduction with dabigatran vs. standard therapy (5.6% vs. 8.8%; HR = 0.63; p = 0.002)
							Major bleeding: similar incidence (1.6% vs. 1.9%; HR = 0.82; p = N/S)

Clinically relevant bleeding defined as the composite of major and non-major clinically relevant bleeding. ^a^30 mg once daily in patients with creatinine clearance 30–50 ml/min or body weight ≤60 kg or in patients receiving concomitant potent P-glycoprotein inhibitors; ^b^Similar outcomes reported in RE-COVER II (data not shown).

bid, twice daily; DVT, deep vein thrombosis; HR, hazard ratio; INR, international normalised ratio; N/S, not specified; od, once daily; PE, pulmonary embolism; RR, relative risk; VKA, vitamin K antagonist; VTE, venous thromboembolism.

EINSTEIN DVT and EINSTEIN PE are notable among phase III trials of direct oral anticoagulants for acute VTE because patients with PE were investigated in a separate study. The rationale for this was the distinct nature and potential severity of PE compared with DVT alone.

There is still a debate regarding the optimal duration of anticoagulant treatment to prevent recurrent VTE. Extended treatment with rivaroxaban has been compared with placebo for the secondary prevention of VTE (Table 3). The double-blind EINSTEIN EXT study enrolled patients who had completed 6–12 months of successful anticoagulation with rivaroxaban or standard therapy and for whom the decision whether to continue or stop anticoagulation was uncertain [4]. Patients received rivaroxaban 20 mg once daily or matched placebo for a further 6 or 12 months. At the end of extended treatment, rivaroxaban was superior to placebo for the prevention of recurrent VTE (1.3% vs. 7.1%; p < 0.001) and led to a non-significant increase in major bleeding (four events with rivaroxaban vs. none with placebo; p = 0.11) [4]. To investigate further the use of rivaroxaban in long-term secondary prevention, the three-arm EINSTEIN CHOICE study will compare a reduced dose of rivaroxaban (10 mg once daily) with a standard dose (20 mg once daily) and with aspirin (100 mg/day) in patients with confirmed symptomatic DVT or PE who have previously completed 6 or 12 months of anticoagulant treatment (NCT02064439).

**Table 3:** **Summary of phase III studies of direct oral anticoagulants for long-term prevention of recurrent VTE** [4, 10, 11]

Drug	Study	Indication	Regimen	Dose schedule	Comparator	Efficacy	Safety
Rivaroxaban	EINSTEIN EXT	Patients with previous VTE already treated for 6–12 months with anticoagulant therapy	Single drug	20 mg od for a further 6 or 12 months	Placebo	Recurrent VTE: rivaroxaban superior to placebo (1.3% vs. 7.1%; HR = 0.18; p < 0.001)	Clinically relevant bleeding: greater incidence with rivaroxaban than placebo (6.0% vs. 1.2%; HR = 5.19; p < 0.001)
							Major bleeding: similar incidence (0.7% vs. 0%; HR N/A; p = 0.11)
Apixaban	AMPLIFY-EXT	Patients with previous VTE already treated for 6–12 months with anticoagulant therapy	Single drug	2.5 mg or 5 mg bid for a further 12 months	Placebo	Recurrent VTE: apixaban superior to placebo (3.8% and 4.2% vs. 11.6%; RR = 0.33 and 0.36; p < 0.001 for both doses)	Clinically relevant bleeding: similar incidence (3.2% and 4.3% vs. 2.7%; RR = 1.20 and RR = 1.62; p = N/S)
							Major bleeding: similar incidence (0.2% and 0.1% vs. 0.5%; RR = 0.49 and RR = 0.25; p = N/S)
Dabigatran	RE-SONATE	Patients with previous VTE already treated for at least 3 months with anticoagulant therapy	Single drug	150 mg bid for a further 6 months	Placebo	Recurrent VTE: dabigatran superior to placebo (0.4% vs. 5.6%; HR = 0.08; p < 0.001)	Clinically relevant bleeding: greater incidence with dabigatran than placebo (5.3% vs. 1.8%; HR = 2.92; p = 0.001)
							Major bleeding: similar incidence (0.3% vs. 0%; HR N/A; p = 1.0)
Dabigatran	RE-MEDY	Patients with previous VTE already treated for at least 3 months with anticoagulant therapy	Single drug	150 mg bid for a further 6 months	Warfarin (INR 2.0–3.0)	Recurrent VTE: dabigatran non-inferior to warfarin (1.8% vs. 1.3%; HR = 1.44; p = 0.01 for non-inferiority)	Clinically relevant bleeding: significantly lower incidence with dabigatran vs. warfarin (5.6% vs. 10.2%; HR = 0.54; p < 0.001)
							Major bleeding: lower incidence with dabigatran but not statistically significant (0.9% vs. 1.8%; HR = 0.52; p = 0.06)

bid, twice daily; HR, hazard ratio; INR, international normalised ratio; N/A, not applicable; N/S, not specified; od, once daily; RR, relative risk; VTE, venous thromboembolism.

## Randomised phase III studies of other direct oral anticoagulants for treatment of venous thromboembolism

Phase III studies of the direct Factor Xa inhibitors apixaban and edoxaban, and the direct thrombin inhibitor dabigatran, all enrolled mixed VTE populations. As with rivaroxaban, apixaban was investigated as a single-drug approach for the treatment of DVT and/or PE in the AMPLIFY study, in which patients were randomised to apixaban 10 mg twice daily for 7 days followed by 5 mg twice daily, or standard enoxaparin/warfarin therapy, for a fixed treatment duration of 6 months (Table 2) [6]. Apixaban was non-inferior to standard therapy for the prevention of recurrent VTE (p < 0.001 for non-inferiority) and led to significant reductions in major bleeding (69% RRR) and clinically relevant bleeding (52% RRR). Edoxaban and dabigatran were tested against standard therapy in the Hokusai-VTE and RE-COVER studies, respectively, but, unlike rivaroxaban and apixaban, they were investigated as part of a dual-drug regimen with initial parenteral anticoagulation (Table 2). Edoxaban was given at a dose of 60 mg once daily (or 30 mg once daily in patients with a CrCl of 30–50 ml/min, a body weight of ≤60 kg or in the case of concomitant treatment with strong P-gp inhibitors). Patients received treatment for between 3 and 12 months based on clinical judgement and patient preference [7]. In RE-COVER and RE-COVER II, dabigatran was given at a fixed 150 mg twice-daily dose and the treatment duration was fixed at 6 months [8, 9]. Both edoxaban and dabigatran were non-inferior to VKA after parenteral induction in these trials (p < 0.001 for non-inferiority), with significant reductions in clinically relevant bleeding and similar rates of major bleeding to conventional treatment [7–9]. Dabigatran is now approved for VTE treatment in the European Union and United States.

Extended treatment with apixaban and dabigatran has also been compared with placebo for the secondary prevention of VTE (Table 3). The AMPLIFY-EXT study compared extended apixaban (2.5 mg or 5 mg twice daily) with placebo in patients who had completed between 6 and 12 months of anticoagulation [10]. After 12 months of further treatment, apixaban was superior to placebo for the prevention of VTE (p < 0.001 for both apixaban doses) and led to a minimal rate of major bleeding (<0.3%). Dabigatran is unique in this setting in that it was compared both against placebo (RE-SONATE) and against warfarin (RE-MEDY) for 6 months of extended treatment in patients who had already received at least 3 months’ anticoagulation [11]. Dabigatran was superior to placebo (p < 0.001) and equivalent to warfarin (p = 0.01 for non-inferiority) for long-term VTE prevention, and was associated with non-significant differences in the rates of major bleeding compared with the comparator treatments. However, there was a significant increase in acute coronary syndromes with dabigatran compared with warfarin (0.9% vs. 0.2%; p = 0.02) [11].

## Practical considerations with rivaroxaban treatment for venous thromboembolism

Rivaroxaban enables acute treatment of DVT and PE (provided the patient is haemodynamically stable) without the need to induce rapid anticoagulation by use of a parenteral agent [4, 5]. This single-drug approach was also tested successfully with apixaban [6], whereas dabigatran and edoxaban were given after heparin in phase III trials [7–9]. With rivaroxaban, the acute treatment phase (15 mg twice daily for 21 days) is followed by once-daily dosing (20 mg) for the prevention of VTE recurrence; this regimen can be continued for as long as judged necessary (even indefinitely in those patients who need lifelong anticoagulation) [12, 13]. However, appropriate communication to primary care and other secondary care providers with regard to the importance of timely transition from twice-daily to once-daily dosing after 3 weeks is essential, as is regular patient contact and education on the importance of correctly adhering to therapy. Ideally, patients should receive a patient card with key information both for them and for healthcare providers. Each manufacturer has their own patient card, whereas an example generic card was suggested in the European Heart Rhythm Association practical guide to direct oral anticoagulants (available to download at http://www.NOACforAF.eu) [45]. Although the guide refers to management of patients with atrial fibrillation, the suggested card can be adapted to other thromboembolic indications for which direct oral anticoagulants may be prescribed.

In general, patients in need of anticoagulation after VTE are younger and have fewer co-morbidities than patients with atrial fibrillation [16]. However, label restrictions apply in case of impaired renal or hepatic function and necessary co-medications. There are also several other practical considerations for rivaroxaban treatment in this indication, derived from the pharmacokinetic evaluation of rivaroxaban.

### Co-morbidities

In patients with CrCl <50 ml/min, the initial rivaroxaban dose of 15 mg twice daily needs no adjustment, and this is also routinely the case for the 20 mg once-daily maintenance dose [12, 13]. However, a reduction in the latter to 15 mg once daily can be considered when the risk of bleeding is judged to outweigh the risk of VTE, although this advice applies only in the European Union and not in the United States [12, 13]. Circumstances in which reduction of the maintenance dose might be considered include, for example, elderly, fragile patients or those requiring additional platelet inhibition using aspirin. It should, nevertheless, be noted that fragile patients in the EINSTEIN DVT and EINSTEIN PE pooled analysis, i.e. those who were elderly, had low body weight or impaired renal function, had similar outcomes without dose reduction compared with the overall patient population, including a significant reduction in major bleeding compared with standard therapy [44]. It should furthermore be noted that rivaroxaban is currently not recommended in children or adolescents (<18 years of age) because of limited clinical data, and it should not be used in pregnant or breastfeeding women [12, 13]. A study on the use of rivaroxaban in the treatment of VTE in children and adolescents aged 6–17 years is ongoing (EINSTEIN JUNIOR, NCT01684423).

In Europe, rivaroxaban is not recommended in patients with CrCl <15 ml/min (<30 ml/min in the United States) or end-stage renal disease [12, 13]. However, in these cases, other oral anticoagulants are also not permitted and choice of treatment is decided on a case-by-case basis; in these patients, UFH treatment is often initiated, followed by VKA [1, 2]. Rivaroxaban is contraindicated in patients with hepatic disease associated with coagulopathy and clinically relevant bleeding risk, including cirrhotic patients classified as Child–Pugh B and C [12, 13]. Before initiating rivaroxaban, an appropriate screen for clinical chemistry parameters, including hepatic function, is necessary; in the opinion of this author, it seems advisable to repeat the test in case of emergent co-morbidities or if the treatment period exceeds 6 months.

### Drug–drug interactions

Rivaroxaban is metabolised by several CYP450 enzymes and is a substrate of CYP3A4 and P-gp [12, 25]. The extent of any interaction between rivaroxaban and other medications is greatly enhanced when a P-gp inhibitor also has CYP450-inhibiting properties. Therefore, co­administration of rivaroxaban with strong inhibitors of both CYP3A4 and P-gp is not recommended [12, 25]; however, the agents concerned, such as systemic antimycotics or some antiretroviral agents, are quite seldom indicated in the ‘normal’ DVT patient population. Medications that are weaker inhibitors of both CYP3A4 and P-gp or strong inhibitors of only one of these pathways have a lesser effect on rivaroxaban pharmacology [12, 25]. Strong inducers of CYP3A4 (e.g. rifampicin or St John’s wort) should be co-administered with caution. Furthermore, rivaroxaban should not be co-administered with dronedarone owing to limited clinical data [12, 13]. As with all direct oral anticoagulants, co-administration with other anticoagulants is not recommended, and caution should be used when non-steroidal anti-inflammatory drugs and agents associated with an elevated risk of bleeding are co-administered [12, 13]. If indicated for coronary artery disease, the use of aspirin at a dose not exceeding 100 mg/day may be combined with rivaroxaban [46], although in patients with stable coronary artery disease, rivaroxaban could be given alone without aspirin, decided on a case-by-case basis [45]. A summary of co-medications to be avoided during treatment with rivaroxaban, and those that may be routinely co-administered or co-administered with caution, is shown in Table 4[12, 13, 25, 47–50].

**Table 4:** **Summary of pharmacokinetic and pharmacodynamic drug interactions with rivaroxaban** [12, 13, 25, 47–50]

Medication class and drug name	Extent of drug–drug interaction with rivaroxaban		
	None/not clinically relevant (routine co-administration possible)	Moderate clinical relevance (co-administer with caution)	High clinical relevance (avoid co-administration)
HIV protease inhibitors			
Ritonavir			X
Azole-antimycotics			
Ketoconazole			X
Fluconazole		X	
Antibiotics			
Clarithromycin	X		
Erythromycin	X		
Rifampicin		X	
Anticoagulants			
Enoxaparin			X
Warfarin			X^a^
NSAIDs and platelet inhibitors			
Naproxen		X	
Clopidogrel		X	
Aspirin		X	
Cardiac medications			
Digoxin	X		
Atorvastatin	X		
Dronedarone			X^b^
Stomach acid regulators			
Ranitidine	X		
Aluminium-magnesium hydroxide (antacid)	X		
Sedatives			
Midazolam	X		
Antidepressants			
St John’s wort		X	
Anticonvulsants			
Phenytoin		X	
Carbamazepine		X	
Phenobarbital		X	

^a^Except when switching; ^b^based on a lack of clinical information.

HIV, human immunodeficiency virus; NSAID, non-steroidal anti-inflammatory drug.

### Switching from or to another anticoagulant

If a patient is receiving a VKA and has suboptimal INR control, switching to rivaroxaban for treatment of VTE or prevention of recurrence is possible by discontinuing the VKA (irrespective of which one has been used) and starting rivaroxaban when the INR is ≤2.5 [12, 13]. There are circumstances in which oral anticoagulation is not possible or adequate in the emergency setting – for example, when the patient is unable to swallow a tablet. In this case, the rivaroxaban tablet may be crushed and given through a gastric tube after confirmation that the tube has been placed correctly [12]. Alternatively, initial parenteral anticoagulation, preferably with LMWH, remains appropriate for induction therapy. It is then possible to switch to rivaroxaban by giving the first dose 0–2 hours before the next dose of LMWH or fondaparinux is scheduled; if UFH has been given as a continuous infusion, the first rivaroxaban dose can be given immediately after termination of the infusion [12, 13]. If the patient is receiving rivaroxaban and parenteral anticoagulation with LMWH becomes necessary (e.g. patient is unable to swallow), the first dose of the LMWH should be given at the time the next rivaroxaban dose would have been scheduled. If rivaroxaban needs to be replaced by another direct oral anticoagulant, the first dose of the other oral agent should be given at the time the next rivaroxaban dose would have been scheduled. If transition from rivaroxaban to a VKA becomes necessary, initiation of the VKA should be effected as normal (loading dose) and rivaroxaban should be co-administered with VKA until the INR is ≥2.0 [12, 13]. The INR should be measured at the time of trough rivaroxaban concentration to minimise interference by rivaroxaban-mediated anticoagulation [51].

If emergency surgery is required in patients receiving rivaroxaban, rivaroxaban should ideally be stopped at least 24 hours before the intervention, taking into account the patient’s risk of bleeding against the potential benefit of surgery [12]. Owing to its short half-life, rivaroxaban can be stopped closer to the time of surgery than VKAs and can then be restarted when post-surgical haemostasis is established. In contrast to VKAs, the rapid onset of anticoagulant effect of rivaroxaban obviates any need for LMWH bridging [52].

### Monitoring

In usual practice, and in common with the other direct oral anticoagulants, rivaroxaban requires no routine monitoring of its anticoagulant effect. However, clinical experience with the new agents has shown that, in certain situations, information about haemostatic status seems valuable, e.g. when the patient is found unconscious, or when a thrombotic or bleeding event is suspected and emergency surgery or invasive procedures are to be considered. Other situations include cases of overdose when the decline of effect or the efficacy of elimination measures needs to be observed, or when making decisions about emergency surgery associated with a high risk of bleeding. In some cases, adherence might also be assessed by a coagulation test. Most laboratory tests are either specific (e.g. calibrated anti-Factor Xa chromogenic assays for rivaroxaban from Diagnostica-Stago, Asnières-sur-Seine, France; Hyphen-BioMed, Neuville-sur-Oise, France; and Technoclone, Vienna, Austria) but not widely available, or are readily available (e.g. prothrombin time or activated partial thromboplastin time) but can provide only qualitative information on overall coagulation [51]. If a specific anti-Factor Xa chromogenic assay is used, expected trough values (24 hours after last dose) for rivaroxaban with the 20 mg once-daily schedule are approximately 50 ng/ml, whereas peak values 2–4 hours after tablet intake are approximately 250 ng/ml (Figure 3) [16]. However, interpretation of such results strongly relies on patient characteristics and an exact knowledge of the dosing history, and the results can be misleading when this information is lacking, for example in cases of impaired patient consciousness. For qualitative measurement (when an anti-Factor Xa assay is not available), the prothrombin time has the best potential to reflect rivaroxaban activity, but a certain therapeutic range cannot be given. More importantly, reagents that are sensitive to rivaroxaban should be used and the reading must be made in seconds; it must not be converted to an INR [52].

### Management of bleeding

The relatively short half-life and the reversible inhibition of Factor Xa by rivaroxaban (bypassing the need for new synthesis of coagulation factors) mean that minor bleeding can often be controlled by temporary or permanent discontinuation of therapy. Recently published guidance on emergency management of bleeding in patients receiving direct oral anticoagulants suggests routine supportive care; activated charcoal may be considered if ingestion was within approximately 2 hours prior to the bleeding event [53–55]. The use of prothrombin complex concentrate, activated prothrombin complex concentrate or recombinant Factor VIIa is potentially useful [12, 56]. Recently, modified recombinant Factor Xa has been investigated as a potential reversal agent for Factor Xa inhibitors [57]. This protein lacks catalytic and membrane-binding activity, but retains the ability to bind Factor Xa inhibitors with subnanomolar affinity, and has the potential to be a universal reversal agent for small-molecule direct Factor Xa inhibitors and antithrombin-dependent indirect Factor Xa inhibitors [58]. This agent is currently undergoing a phase II clinical study in healthy volunteers (NCT01758432), but it is not expected to be commercially available before 2016.

### Administration

The rivaroxaban doses approved for the treatment of VTE should be taken with a meal; tablets may be disintegrated and mixed with food to facilitate swallowing [12, 13]. During the acute treatment phase (15 mg twice daily), if the patient misses a morning dose, they should be instructed to take this missing dose immediately (if necessary at the same time as the later dose) to ensure the required 30 mg total daily dose. A missed morning dose during the maintenance phase (20 mg once daily) should be taken within the day, but not added to the next day’s dose [12, 13].

## Conclusions

Rivaroxaban has predictable pharmacokinetic properties and exhibits rapid-onset, dose-proportional pharmacodynamic effects that make it suitable for use as a single-drug anticoagulant treatment for patients with VTE. The approved rivaroxaban regimen for VTE treatment (15 mg twice daily followed by 20 mg once daily) was derived based on dose-finding studies and pharmacokinetic modelling, and balances the need for a strong antithrombotic effect in the acute phase of VTE treatment with a once-daily maintenance dose with a favourable benefit–risk profile. Clinical data from randomised phase III studies support the use of this regimen. Rivaroxaban is suitable for a wide range of patients, but consideration should be given to renal and hepatic function and co-medications, as well as other practical aspects, before and during treatment.

## Abbreviations

AUC: Area under the concentration–time curve
C_max_: Maximum plasma concentration
CrCl: Creatinine clearance
CYP: Cytochrome P450
DVT: Deep vein thrombosis
HIV: Human immunodeficiency virus
INR: International normalised ratio
LMWH: Low molecular weight heparin
P-gp: P-glycoprotein
PE: Pulmonary embolism
RRR: Relative risk reduction
UFH: Unfractionated heparin
VKA: Vitamin K antagonist
VTE: Venous thromboembolism.

## References

[1] Torbicki A, Perrier A, Konstantinides S, Agnelli G, Galiè N, Pruszczyk P, Bengel F, Brady AJ, Ferreira D, Janssens U, Klepetko W, Mayer E, Remy-Jardin M, Bassand JP (2008). Guidelines on the diagnosis and management of acute pulmonary embolism: the Task Force for the Diagnosis and Management of Acute Pulmonary Embolism of the European Society of Cardiology (ESC). Eur Heart J.

[2] Kearon C, Akl EA, Comerota AJ, Prandoni P, Bounameaux H, Goldhaber SZ, Nelson ME, Wells PS, Gould MK, Dentali F, Crowther M, Kahn SR (2012). Antithrombotic therapy for VTE disease: antithrombotic therapy and prevention of thrombosis, 9th ed: American College of Chest Physicians evidence-based clinical practice guidelines. Chest.

[3] Eikelboom JW, Weitz JI (2010). New anticoagulants. Circulation.

[4] The EINSTEIN Investigators (2010). Oral rivaroxaban for symptomatic venous thromboembolism. N Engl J Med.

[5] The EINSTEIN–PE Investigators (2012). Oral rivaroxaban for the treatment of symptomatic pulmonary embolism. N Engl J Med.

[6] Agnelli G, Buller HR, Cohen A, Curto M, Gallus AS, Johnson M, Masiukiewicz U, Pak R, Thompson J, Raskob GE, Weitz JI, AMPLIFY Investigators (2013). Oral apixaban for the treatment of acute venous thromboembolism. N Engl J Med.

[7] The Hokusai-VTE Investigators (2013). Edoxaban versus warfarin for the treatment of symptomatic venous thromboembolism. N Engl J Med.

[8] Schulman S, Kearon C, Kakkar AK, Mismetti P, Schellong S, Eriksson H, Baanstra D, Schnee J, Goldhaber SZ, for the RE-COVER Study Group (2009). Dabigatran versus warfarin in the treatment of acute venous thromboembolism. N Engl J Med.

[9] Schulman S, Kakkar AK, Goldhaber SZ, Schellong S, Eriksson H, Mismetti P, Vedel Christiansen A, Friedman J, Le Maulf F, Peter N, Kearon C, RE-COVER II Trial Investigators (2014). Treatment of acute venous thromboembolism with dabigatran or warfarin and pooled analysis. Circulation.

[10] Agnelli G, Buller HR, Cohen A, Curto M, Gallus AS, Johnson M, Porcari A, Raskob GE, Weitz JI, AMPLIFY-EXT Investigators (2013). Apixaban for extended treatment of venous thromboembolism. N Engl J Med.

[11] Schulman S, Kearon C, Kakkar AK, Schellong S, Eriksson H, Baanstra D, Kvamme AM, Friedman J, Mismetti P, Goldhaber SZ (2013). Extended use of dabigatran, warfarin, or placebo in venous thromboembolism. N Engl J Med.

[12] Bayer Pharma AG (2014). Xarelto® (rivaroxaban) Summary of Product Characteristics.

[13] Janssen Pharmaceuticals Inc (2014). Xarelto® (rivaroxaban) Prescribing Information.

[14] Agnelli G, Gallus A, Goldhaber SZ, Haas S, Huisman MV, Hull RD, Kakkar AK, Misselwitz F, Schellong S, ODIXa-DVT Study Investigators (2007). Treatment of proximal deep-vein thrombosis with the oral direct Factor Xa inhibitor rivaroxaban (BAY 59–7939): the ODIXa-DVT (Oral Direct Factor Xa Inhibitor BAY 59–7939 in patients with acute symptomatic Deep-Vein Thrombosis) study. Circulation.

[15] Buller HR, Lensing AWA, Prins MH, Agnelli G, Cohen A, Gallus AS, Misselwitz F, Raskob G, Schellong S, Segers A (2008). A dose-ranging study evaluating once-daily oral administration of the Factor Xa inhibitor rivaroxaban in the treatment of patients with acute symptomatic deep vein thrombosis: the Einstein-DVT Dose-Ranging Study. Blood.

[16] Mueck W, Lensing AWA, Agnelli G, Décousus H, Prandoni P, Misselwitz F (2011). Rivaroxaban: population pharmacokinetic analyses in patients treated for acute deep-vein thrombosis and exposure simulations in patients with atrial fibrillation treated for stroke prevention. Clin Pharmacokinet.

[17] Perzborn E, Strassburger J, Wilmen A, Pohlmann J, Roehrig S, Schlemmer KH, Straub A (2005). *In vitro* and *in vivo* studies of the novel antithrombotic agent BAY 59–7939 - an oral, direct Factor Xa inhibitor. J Thromb Haemost.

[18] Kubitza D, Becka M, Voith B, Zuehlsdorf M, Wensing G (2005). Safety, pharmacodynamics, and pharmacokinetics of single doses of BAY 59–7939, an oral, direct Factor Xa inhibitor. Clin Pharmacol Ther.

[19] Perzborn E, Roehrig S, Straub A, Kubitza D, Misselwitz F (2011). The discovery and development of rivaroxaban, an oral, direct Factor Xa inhibitor. Nat Rev Drug Discov.

[20] Weinz C, Schwarz T, Kubitza D, Mueck W, Lang D (2009). Metabolism and excretion of rivaroxaban, an oral, direct Factor Xa inhibitor, in rats, dogs and humans. Drug Metab Dispos.

[21] Ufer M (2010). Comparative efficacy and safety of the novel oral anticoagulants dabigatran, rivaroxaban and apixaban in preclinical and clinical development. Thromb Haemost.

[22] Camm AJ, Bounameaux H (2011). Edoxaban: a new oral direct Factor Xa inhibitor. Drugs.

[23] Eriksson BI, Quinlan DJ, Eikelboom JW (2011). Novel oral Factor Xa and thrombin inhibitors in the management of thromboembolism. Annu Rev Med.

[24] Harder S (2012). Renal profiles of anticoagulants. J Clin Pharmacol.

[25] Mueck W, Kubitza D, Becka M (2013). Co-administration of rivaroxaban with drugs that share its elimination pathways: pharmacokinetic effects in healthy subjects. Br J Clin Pharmacol.

[26] Kubitza D, Becka M, Wensing G, Voith B, Zuehlsdorf M (2005). Safety, pharmacodynamics, and pharmacokinetics of BAY 59–7939 – an oral, direct Factor Xa inhibitor – after multiple dosing in healthy male subjects. Eur J Clin Pharmacol.

[27] Gerotziafas GT, Elalamy I, Depasse F, Perzborn E, Samama MM (2007). *In vitro* inhibition of thrombin generation, after tissue factor pathway activation, by the oral, direct Factor Xa inhibitor rivaroxaban. J Thromb Haemost.

[28] Graff J, von Hentig N, Misselwitz F, Kubitza D, Becka M, Breddin HK, Harder S (2007). Effects of the oral, direct Factor Xa inhibitor rivaroxaban on platelet-induced thrombin generation and prothrombinase activity. J Clin Pharmacol.

[29] Kubitza D, Becka M, Zuehlsdorf M, Mueck W (2007). Body weight has limited influence on the safety, tolerability, pharmacokinetics, or pharmacodynamics of rivaroxaban (BAY 59–7939) in healthy subjects. J Clin Pharmacol.

[30] Kubitza D, Becka M, Roth A, Mueck W (2013). The influence of age and gender on the pharmacokinetics and pharmacodynamics of rivaroxaban – an oral, direct Factor Xa inhibitor. J Clin Pharmacol.

[31] Jiang J, Hu Y, Zhang J, Yang J, Mueck W, Kubitza D, Bauer RJ, Meng L, Hu P (2010). Safety, pharmacokinetics and pharmacodynamics of single doses of rivaroxaban - an oral, direct Factor Xa inhibitor - in elderly Chinese subjects. Thromb Haemost.

[32] Zhao X, Sun P, Zhou Y, Liu Y, Zhang H, Mueck W, Kubitza D, Bauer RJ, Zhang H, Cui Y (2009). Safety, pharmacokinetics and pharmacodynamics of single/multiple doses of the oral, direct Factor Xa inhibitor rivaroxaban in healthy Chinese subjects. Br J Clin Pharmacol.

[33] Stampfuss J, Kubitza D, Becka M, Mueck W (2013). The effect of food on the absorption and pharmacokinetics of rivaroxaban. Int J Clin Pharmacol Ther.

[34] Kubitza D, Becka M, Mueck W, Halabi A, Maatouk H, Klause N, Lufft V, Wand DD, Philipp T, Bruck H (2010). Effects of renal impairment on the pharmacokinetics, pharmacodynamics and safety of rivaroxaban, an oral, direct Factor Xa inhibitor. Br J Clin Pharmacol.

[35] Kubitza D, Becka M, Schwers S, Voith B (2013). Investigation of pharmacodynamic and pharmacokinetic interactions between rivaroxaban and enoxaparin in healthy male subjects. Clinical Pharm in Drug Dev.

[36] Kubitza D, Becka M, Mück W, Krätzschmar J (2014). Pharmacodynamics and pharmacokinetics during the transition from warfarin to rivaroxaban: a randomized study in healthy subjects. Br J Clin Pharmacol.

[37] Kubitza D, Roth A, Becka M, Alatrach A, Halabi A, Hinrichsen H, Mueck W (2013). Effect of hepatic impairment on the pharmacokinetics and pharmacodynamics of a single dose of rivaroxaban – an oral, direct Factor Xa inhibitor. Br J Clin Pharmacol.

[38] Fiessinger JN, Huisman MV, Davidson BL, Bounameaux H, Francis CW, Eriksson H, Lundström T, Berkowitz SD, Nyström P, Thorsén M, Ginsberg JS, THRIVE Treatment Study Investigators (2005). Ximelagatran vs low-molecular-weight heparin and warfarin for the treatment of deep vein thrombosis: a randomized trial. JAMA.

[39] van Gogh Investigators (2007). Idraparinux versus standard therapy for venous thromboembolic disease. N Engl J Med.

[40] Laliberté F, Bookhart BK, Nelson WW, Lefebvre P, Schein JR, Rondeau-Leclaire J, Duh MS (2013). Impact of once-daily versus twice-daily dosing frequency on adherence to chronic medications among patients with venous thromboembolism. Patient.

[41] Mueck W, Becka M, Kubitza D, Voith B, Zuehlsdorf M (2007). Population model of the pharmacokinetics and pharmacodynamics of rivaroxaban - an oral, direct Factor Xa inhibitor - in healthy subjects. Int J Clin Pharmacol Ther.

[42] Mueck W, Eriksson BI, Bauer KA, Borris L, Dahl OE, Fisher WD, Gent M, Haas S, Huisman MV, Kakkar AK, Kälebo P, Kwong LM, Misselwitz F, Turpie AGG (2008). Population pharmacokinetics and pharmacodynamics of rivaroxaban – an oral, direct Factor Xa inhibitor – in patients undergoing major orthopaedic surgery. Clin Pharmacokinet.

[43] Mueck W, Borris LC, Dahl OE, Haas S, Huisman MV, Kakkar AK, Kälebo P, Muelhofer E, Misselwitz F, Eriksson BI (2008). Population pharmacokinetics and pharmacodynamics of once- and twice-daily rivaroxaban for the prevention of venous thromboembolism in patients undergoing total hip replacement. Thromb Haemost.

[44] Prins MH, Lensing AWA, Bauersachs R, van Bellen B, Bounameaux H, Brighton TA, Cohen AT, Davidson BL, Decousus H, Raskob GE, Berkowitz SD, Wells PS, on behalf of the EINSTEIN Investigators (2013). Oral rivaroxaban versus standard therapy for the treatment of symptomatic venous thromboembolism: a pooled analysis of the EINSTEIN-DVT and PE randomized studies. Thromb J.

[45] Heidbuchel H, Verhamme P, Alings M, Antz M, Hacke W, Oldgren J, Sinnaeve P, Camm AJ, Kirchhof P (2013). European Heart Rhythm Association Practical Guide on the use of new oral anticoagulants in patients with non-valvular atrial fibrillation. Europace.

[46] Turpie AGG, Kreutz R, Llau J, Norrving B, Haas S (2012). Management consensus guidance for the use of rivaroxaban - an oral, direct Factor Xa inhibitor. Thromb Haemost.

[47] Kubitza D, Becka M, Mueck W, Zuehlsdorf M (2006). Safety, tolerability, pharmacodynamics, and pharmacokinetics of rivaroxaban – an oral, direct Factor Xa inhibitor – are not affected by aspirin. J Clin Pharmacol.

[48] Kubitza D, Becka M, Zuehlsdorf M, Mueck W (2006). Effect of food, an antacid, and the H2 antagonist ranitidine on the absorption of BAY 59–7939 (rivaroxaban), an oral, direct Factor Xa inhibitor, in healthy subjects. J Clin Pharmacol.

[49] Kubitza D, Becka M, Mueck W, Zuehlsdorf M (2007). Rivaroxaban (BAY 59–7939) - an oral, direct Factor Xa inhibitor - has no clinically relevant interaction with naproxen. Br J Clin Pharmacol.

[50] Kubitza D, Becka M, Roth A, Mueck W (2012). Absence of clinically relevant interactions between rivaroxaban - an oral, direct Factor Xa inhibitor - and digoxin or atorvastatin in healthy subjects. J Int Med Res.

[51] Lindhoff-Last E, Ansell J, Spiro T, Samama MM (2013). Laboratory testing of rivaroxaban in routine clinical practice: when, how, and which assays. Ann Med.

[52] Goldstein P, Elalamy I, Huber K, Danchin N, Wiel E (2013). Rivaroxaban and other non-vitamin K antagonist oral anticoagulants in the emergency treatment of thromboembolism. Int J Emerg Med.

[53] Kaatz S, Kouides PA, Garcia DA, Spyropolous AC, Crowther M, Douketis JD, Chan AK, James A, Moll S, Ortel TL, Van Cott EM, Ansell J (2012). Guidance on the emergent reversal of oral thrombin and Factor Xa inhibitors. Am J Hematol.

[54] Alikhan R, Rayment R, Keeling D, Baglin T, Benson G, Green L, Marshall S, Patel R, Pavord S, Rose P, Tait C (2014). The acute management of haemorrhage, surgery and overdose in patients receiving dabigatran. Emerg Med J.

[55] Peacock WF, Gearhart MM, Mills RM (2012). Emergency management of bleeding associated with old and new oral anticoagulants. Clin Cardiol.

[56] Perzborn E, Heitmeier S, Laux V, Buchmuller A (2014). Reversal of rivaroxaban-induced anticoagulation with prothrombin complex concentrate, activated prothrombin complex concentrate and recombinant activated factor VII in vitro. Thromb Res.

[57] Lu G, DeGuzman FR, Hollenbach SJ, Karbarz MJ, Abe K, Lee G, Luan P, Hutchaleelaha A, Inagaki M, Conley PB, Phillips DR, Sinha U (2013). A specific antidote for reversal of anticoagulation by direct and indirect inhibitors of coagulation Factor Xa. Nat Med.

[58] Siegal DM, Cuker A (2014). Reversal of target-specific oral anticoagulants. Drug Discov Today.

